# Healthy sleep pattern is associated with reduced risk of incident thyroid disease: a prospective cohort study from the UK Biobank

**DOI:** 10.3389/fendo.2026.1859828

**Published:** 2026-10-02

**Authors:** Jingxue Sun, Yanmeizhi Wu, Xiaoyu Zhao, Lulu Sun, Huanran Lv, Ruitong Sun, Tianshu Han, Hong Qiao

**Affiliations:** 1 Department of Endocrinology and Metabolism, The Second Affiliated Hospital of Harbin Medical University, Harbin, China; 2 Department of Nutrition and Food Hygiene, School of Public Health, the National Key Discipline, Harbin Medical University, Harbin, China; 3 Key Laboratory of Precision Nutrition and Health, Ministry of Education, Harbin Medical University, Harbin, China

**Keywords:** hypothyroidism, sleep pattern, thyroid, thyrotoxicosis, UK Biobank

## Abstract

**Introduction:**

The longitudinal association between sleep behaviors and the incidence of various thyroid diseases remains unclear. This study aimed to prospectively investigate the association between a healthy sleep score and the risk of incident thyroid diseases using data from the UK Biobank.

**Methods:**

This prospective cohort study included 499, 084 adults aged 37–73 years from the UK Biobank. A healthy sleep score ranging from 0 to 5 was constructed based on five self-reported sleep behaviors: chronotype, sleep duration, insomnia, snoring, and daytime excessive sleepiness. Participants with missing information on sleep behaviors were excluded from the construction of the healthy sleep score. Incident outcomes included overall thyroid disease, hypothyroidism, nontoxic goiter, thyrotoxicosis, thyroiditis, thyroid cancer, and iodine deficiency–related diseases.

**Results:**

After adjustment for multiple covariates, higher healthy sleep scores were dose-dependently associated with lower risks of overall thyroid disease, hypothyroidism, nontoxic goiter, and thyrotoxicosis. Compared with participants with poor sleep patterns (scores of 0–1), those with the healthiest sleep pattern (score of 5) had significantly lower risks of overall thyroid disease (24% [17%–31%]), hypothyroidism (25% [17%–32%]), nontoxic goiter (26% [7%–41%]), and thyrotoxicosis (32% [15%–45%]). Among the five sleep components, the absence of frequent daytime excessive sleepiness showed a particularly evident association with lower risks of thyroid disease. Furthermore, interaction analyses suggested that the inverse association between the healthy sleep score and the risk of overall thyroid disease may vary by sex, BMI, and diabetes status, with more pronounced associations observed among women, individuals with obesity, and those without diabetes.

**Conclusion:**

Higher healthy sleep scores were associated with lower risks of incident overall thyroid disease, hypothyroidism, nontoxic goiter, and thyrotoxicosis. Among the individual sleep components, the absence of frequent daytime excessive sleepiness showed the strongest association with a lower risk of thyroid disease.

## 1 Introduction

Thyroid diseases comprise a group of disorders that affect thyroid function or structure, including hyperthyroidism, hypothyroidism, thyroiditis, thyroid cancer, goiter, and iodine deficiency–related thyroid diseases (1). The incidence of thyroid diseases has increased rapidly (2). According to estimates from the American Thyroid Association, more than 12% of the U.S. population will develop thyroid disease during their lifetime (3). Most thyroid diseases require long-term or lifelong pharmacological treatment, have relatively high recurrence rates, and substantially impair quality of life (4, 5). Therefore, identifying potentially modifiable risk factors associated with the development of thyroid diseases is an important component of their prevention and management.

Although the etiology of thyroid diseases is relatively complex, disruption of circadian rhythms has been considered an important contributing factor. Accordingly, an increasing number of studies have examined the associations of sleep duration, sleep quality, and chronotype with the prevalence of thyroid diseases, as these sleep-related parameters are reliable proxies for circadian rhythms (6–9). However, these studies were cross-sectional in design and could not exclude the possibility that patients with thyroid diseases had poorer sleep behaviors. Prospective studies are therefore needed to provide more robust and comprehensive evidence regarding the association between sleep behaviors and the incidence of thyroid diseases.

Notably, the potential causal nature of this association has recently been supported by Mendelian randomization studies. Fan et al. reported that genetic predisposition to insomnia and short sleep duration was causally associated with increased risks of thyroid dysfunction and thyroid cancer (10). In addition, sleep behavior is multidimensional, typically encompassing sleep duration and quality, chronotype, and daytime napping (11). Previous cross-sectional studies frequently focused on the association between a single aspect of sleep behavior and the development of thyroid diseases. It remains unclear whether, and how, different dimensions of sleep behavior influence the development of thyroid diseases, although these studies may provide important insights into this question.

Therefore, to address these knowledge gaps, this study aimed to prospectively investigate the associations of sleep duration, chronotype, insomnia, snoring, and daytime excessive sleepiness with the risk of incident thyroid diseases among 499, 084 participants from the UK Biobank.

## 2 Methods

### 2.1 Study population

The UK Biobank is a large-scale prospective cohort study that recruited more than 500, 000 participants aged 37–73 years from 22 assessment centers across England, Scotland, and Wales between 2006 and 2010. Briefly, participants provided a wide range of health-related information, including sociodemographic characteristics, life-course exposures, and medical history, through touchscreen questionnaires and physical measurements conducted at recruitment.

The UK Biobank study was approved by the National Health Service National Information Governance Board for Health and Social Care in England and Wales, the Community Health Index Advisory Group in Scotland, and the North West Multi-centre Research Ethics Committee. All participants provided written informed consent.

In this study, we excluded 3, 327 participants with missing information on sleep behaviors at baseline, leaving 499, 084 participants for the initial analysis. For prospective analyses of overall thyroid disease and specific thyroid disease outcomes, participants with the corresponding disease at baseline were further excluded, and separate risk sets were constructed for each outcome. Prevalent cases at baseline were identified according to disease diagnosis records before or on the date of recruitment.

Among the baseline analytic population, we excluded 78 participants with iodine deficiency–related thyroid diseases and related conditions, 36 with iodine deficiency hypothyroidism, 26, 270 with hypothyroidism, 3, 010 with nontoxic goiter, 5, 404 with thyrotoxicosis, 881 with thyroiditis, 444 with thyroid cancer, and 1, 756 with other thyroid diseases (E07). After these exclusions, Cox proportional hazards regression analyses were conducted among participants free of the corresponding outcome at baseline.

### 2.2 Assessment of sleep behaviors and healthy sleep score

In the UK Biobank, self-reported dimensions of sleep, including sleep duration, chronotype, sleeplessness/insomnia, snoring, and daytime sleepiness, were recorded by touchscreen questionnaires. The participants’ sleep duration was evaluated by asking them the question, “About how many hours sleep including naps do you get in every 24 hours?”. The chronotype was assessed by asking, “Do you consider yourself to be?”. With the following response categories: “definitely a morning person”, “more a morning than evening person”, “more an evening than morning person”, “definitely an evening person”, “do not know”, and “prefer not to answer”. Insomnia was assessed by asking, “Do you have trouble falling asleep at night or do you wake up in the middle of the night?”. The response categories were “never/rarely”, “sometimes”, “usually”, and “prefer not to answer”. Information on snoring was collected through the question, “Does your partner or a close relative or friend complain about your snoring?”. Participants could choose from the response options “yes, “ “no, “ “do not know, “ and “prefer not to answer.” Data on daytime sleeping were collected by asking, “How likely are you to doze off or fall asleep during the daytime when you do not mean to?” The response options included “never/rarely, “ “sometimes, “ “often, “ “do not know, “ “prefer not to answer, “ and “all of the time”. If there was significant variation in sleep behaviors, participants were asked to provide an average answer for the last month.

The well-established sleep behaviors were defined by 5 aspects: 1) 7 to 8 hours of sleep per day, 2) being an early chronotype (morning person or more of a morning person than an evening person), 3) no frequent insomnia (never or rarely), 4) no snoring, and 5) no frequent excessive daytime sleepiness (never/rarely or sometimes). The participants received 1 point for each adherence to these favorable sleep behaviors, while 0 points were assigned if the criteria were not met. The healthy sleep score was calculated as the sum of all 5 individual sleep behaviors. A higher score indicated a healthier sleep score. Because participants with prevalent disease at baseline were excluded separately for each thyroid disease outcome, the analytical sample size and the number of participants with a healthy sleep score of 0 varied slightly across outcome-specific analyses. The number of participants with a score of 0 was small for all outcomes (2, 672–2, 867); therefore, using this group alone as the reference category could yield unstable risk estimates. Accordingly, consistent with a previous study (12), participants with healthy sleep scores of 0 and 1 were combined and treated as the reference group.

### 2.3 Assessment of thyroid diseases

The main outcomes in the present study were overall and specific thyroid diseases, which are determined by the International Classification of Diseases edition 10 (ICD-10), including the iodine-deficiency-related thyroid disorders and allied conditions (E01), iodine-deficiency hypothyroidism(E02), hypothyroidism (E03), non-toxic goiter (E04), thyrotoxicosis(E05), thyroiditis(E06), other disorders of thyroid(E07), thyroid cancer (C73). In addition, we also use ICD-9 code 193 to define thyroid cancer. Thyroid disease is defined as having any of the above diseases. This research has been conducted using the UK Biobank Resource. The follow-up period for each participant is from the recruitment date to February 1, 2022 or the date of participant death, whichever comes first.

### 2.4 Assessment of covariates

The covariates of demographic and lifestyle behaviors were collected at baseline, such as age (years), gender (male, female), ethnic (White, Asian or Asian British, Black or Black British, Chinese, Other ethnic, mixed), income (Less than 18000, 18000 to 31000, 31000 to 52000, 52000 to 100000, Greater than 100000), and education (College or University degree, A levels/AS levels or equivalent, O levels/GCSEs or equivalent, NVQ or HND or HNC or equivalent, Other professional qualifications). Physical activity level (PAL. level) was categorized as low, moderate, and high. Smoking and drinking status included never, previous and current. Height, weight and hip circumference (Hip) were measured in the assessment centers, and BMI was calculated based on weight divided by the square of height (kg/m^2^). Preexisting diabetes was defined as self-reported physician-diagnosed cases.

### 2.5 Statistical analyses

Baseline characteristics were summarized according to categories of the healthy sleep score. Continuous variables are presented as means and standard deviations, whereas categorical variables are presented as frequencies and percentages. Differences across groups were compared using general linear models and chi-square tests.

Multivariable-adjusted Cox proportional hazards regression models were used to estimate hazard ratios (HRs) and 95% confidence intervals (CIs) for the associations between the healthy sleep score and the risk of thyroid diseases, with participants scoring 0–1 as the reference group. Model 1 was adjusted for age, sex, ethnicity, hip circumference, and body mass index (BMI). Model 2 was further adjusted for income, educational attainment, smoking status, alcohol consumption status, and the Townsend deprivation index. Model 3 additionally included physical activity level (PAL) and diabetes. Based on the covariate adjustments in Model 3, Cox proportional hazards regression analyses were also performed to examine the associations between individual sleep behaviors and the risk of incident thyroid diseases. Predefined missing-value codes in the original data were included as separate categories in the regression models. Multiple imputation was not performed for genuine missing values in the remaining covariates. Instead, complete-case analyses were conducted for each Cox proportional hazards regression model, excluding participants with missing data for any variable included in that model. Consequently, the actual analytical sample size may have varied slightly across the different adjustment models.

The stratified the analyses were performed according to: age (<45years, 45–59 years, >60 years), gender (male, female), BMI (18.5-24.9kg/m2, 25-29.9 kg/m2, ≥30 kg/m2), smoking status (never smoking, previous smoking, and current smoking), drinking status (never drinking, previous drinking, and current drinking), and preexisting diabetes (yes, no).

For specific thyroid diseases in which statistically significant associations between the healthy sleep score and outcome risk were observed in subgroup analyses, interactions were further assessed by including cross-product terms between the healthy sleep score and the stratification variables in Cox proportional hazards regression models. Smoking status and alcohol consumption status were each categorized as never, former, or current, and their interactions with the healthy sleep score were examined separately. Interaction analyses were adjusted for the same covariates as in the primary analyses. Statistical significance was defined as a two-sided *P*_interaction_ *<*0.05.

All analyses were performed by SPSS Statistics 26, GraphPad Prism was used for graphing. A two-sided *P* < 0.05 was considered to be statistically significant.

## 3 Results

### 3.1 Baseline characteristics

Baseline characteristics according to healthy sleep scores are presented in **Table 1**. Overall, compared with participants with the healthiest sleep pattern (sleep score of 5), those with poor sleep patterns (sleep scores of 0–1) had a higher prevalence of thyroid diseases, including hypothyroidism, nontoxic goiter, thyrotoxicosis, and other thyroid Vdiseases. Participants with higher healthy sleep scores were more likely to be female and to have lower BMI and hip circumference, higher income and educational attainment, lower levels of deprivation, never-smoking status, and higher levels of physical activity. They also tended to have a lower prevalence of diabetes.

**Table 1:** Baseline characteristics of the study population according to the healthy sleep scores.

Characteristic	Healthy sleep score					*P value*
	0-1	2	3	4	5	
Age [mean(SD)]	56.45 (7.93)	56.78 (7.91)	56.75 (8.02)	56.34 (8.24)	54.97 (8.56)	<0.001
Gender[n(%)]						
Male	16537 (52)	56578 (49)	85540 (45)	55973 (42.3)	13243 (45.4)	<0.001
Female	15236 (48)	58990 (51)	104620 (55)	76462 (57.7)	15902 (54.6)	*-*
Ethnic [n(%)]						
White person	27966 (88)	108216 (93.6)	180212 (94.8)	126077 (95.2)	27493 (94.3)	*<* *0.001*
Other ethnic	3809 (12)	7353 (6.4)	9948 (5.2)	6358 (4.8)	1652 (5.7)	*-*
Hip [mean(SD)]	106.23 (10.78)	104.56 (9.69)	103.3 (9.07)	102.25 (8.57)	101.46 (8.16)	*<* *0.001*
BMI [mean(SD)]	29.4 (5.6)	28.23 (5.02)	27.35 (4.68)	26.64 (4.4)	26.15 (4.2)	*<* *0.001*
Income [n(%)]						
Less than 100000	30988 (97.5)	111431 (96.4)	181575 (95.5)	125142 (94.5)	27028 (92.7)	<0.001
Greater than 100000	787 (2.5)	4138 (3.6)	8585 (4.5)	7293 (5.5)	2117 (7.3)	*-*
Education [n(%)]						
College or university degree	7684 (24.2)	33005 (28.6)	61068 (32.1)	47420 (35.8)	11567 (39.7)	<0.001
Others	24091 (75.8)	82564 (71.4)	129092 (67.9)	85015 (64.2)	17578 (60.3)	*-*
Townsend.index [mean(SD)]	-0.42 (3.45)	-1.06 (3.2)	-1.38 (3.04)	-1.58 (2.93)	-1.6 (2.93)	*<* *0.001*
Smoking status [n(%)]						
Never	14121 (44.4)	57645 (49.9)	103464 (54.4)	78031 (58.9)	18496 (63.5)	<0.001
Previous	11345 (35.7)	41830 (36.2)	66798 (35.1)	43656 (33)	8513 (29.2)	*-*
Current	5226 (16.4)	15577 (13.5)	19235 (10.1)	10368 (7.8)	2058 (7.1)	*-*
Unknown or Prefer not to answer	1083 (3.5)	517 (0.4)	663 (0.4)	380 (0.3)	78 (0.3)	*-*
Drink status [n(%)]						
never	1634 (5.1)	5002 (4.3)	8001 (4.2)	5865 (4.4)	1512 (5.2)	<0.001
previous	1602 (5)	4473 (3.9)	6471 (3.4)	4280 (3.2)	1006 (3.5)	*-*
current	27550 (86.7)	105924 (91.7)	175507 (92.3)	122196 (92.3)	26614 (91.3)	*-*
Unknown or Prefer not to answer	989 (3.2)	170 (0.1)	181 (0.1)	94 (0.1)	13 (0.0)	*-*
PAL level [n(%)]						
Low	6508 (20.5)	20013 (17.3)	28769 (15.1)	17458 (13.2)	3357 (11.5)	<0.001
Moderate	8787 (27.7)	36446 (31.5)	63115 (33.2)	45300 (34.2)	9975 (34.2)	*-*
High	7513 (23.6)	33014 (28.6)	61583 (32.4)	47809 (36.1)	11895 (40.8)	*-*
Other	8967 (28.2)	26096 (22.6)	36693 (19.3)	21868 (16.5)	3918 (13.5)	*-*
Diabetes [n(%)]						
No	28065 (88.3)	106246 (91.9)	178436 (93.8)	125821 (95)	27858 (95.6)	*<* *0.001*
Yes	3710 (11.7)	9323 (8.1)	11724 (6.2)	6614 (5)	1287 (4.4)	*-*

Data are presented as mean (standard deviation) for continuous variables and number (percentage) for categorical variables. Yes : Already occurred at baseline; BMI, body mass index; PAL, physical activity level. Percentages were rounded to one decimal place; values <0.05% were presented as 0.0%.

### 3.2 Association between healthy sleep score and the risk of developing thyroid disease

During a mean follow-up of 15.89 years, 15, 135 participants developed incident thyroid disease, including 12, 537 cases of hypothyroidism, 2, 554 cases of nontoxic goiter, 2, 925 cases of thyrotoxicosis, 518 cases of thyroiditis, 489 cases of thyroid cancer, 25 cases of iodine-deficiency–related thyroid disorders and allied conditions, 152 cases of iodine-deficiency–related hypothyroidism, and 962 cases of other thyroid disorders.

The associations between the healthy sleep score and the risk of incident thyroid diseases are presented in **Table 2**. After adjustment for potential confounders in Model 3, compared with participants with healthy sleep scores of 0–1, those with a score of 5 had lower risks of overall thyroid disease (HR, 0.759; 95% CI, 0.690–0.834), hypothyroidism (HR, 0.749; 95% CI, 0.675–0.832), nontoxic goiter (HR, 0.742; 95% CI, 0.590–0.933), and thyrotoxicosis (HR, 0.680; 95% CI, 0.546–0.847). No significant associations were observed between the healthy sleep score and the risk of incident thyroiditis, thyroid cancer, iodine-deficiency–related thyroid disorders and allied conditions, iodine-deficiency–related hypothyroidism, or other thyroid disorders.

**Table 2:** Association between healthy sleep score and the risk of developing overall and specific thyroid diseases.

Sleep score	Casas/N	Person-years	Model1		Model2		Model3	
			HR (95%CI)	*P value*	HR (95%CI)	*P value*	HR (95%CI)	*P value*
Thyroid disease								
0-1	1221/29396	359198.73	Ref	*<0.001*	Ref	*<0.001*	Ref	*<0.001*
2	3790/107604	1331379.62	0.848 (0.794, 0.906)	*-*	0.876 (0.820, 0.936)	*-*	0.883 (0.827, 0.943)	*-*
3	5716/177723	2210085.54	0.769 (0.721, 0.819)		0.814 (0.764, 0.868)		0.824 (0.773, 0.878)	
4	3687/124175	1551228.59	0.719 (0.673, 0.769)		0.775 (0.725, 0.829)		0.787 (0.735, 0.842)	
5	721/27626	346070.27	0.685 (0.623, 0.752)		0.746 (0.679, 0.82)		0.759 (0.690, 0.834)	
Hypothyroidism								
0-1	1028/29845	382243.47	Ref	*<0.001*	Ref	*<0.001*	Ref	*<0.001*
2	3159/109108	1402870.77	0.849 (0.790, 0.913)	*-*	0.880 (0.819, 0.946)	*-*	0.886 (0.824, 0.952)	*-*
3	4750/180296	2319077.22	0.771 (0.719, 0.827)		0.822 (0.766, 0.881)		0.829 (0.773, 0.889)	
4	3021/125893	1619361.37	0.714 (0.664, 0.768)		0.777 (0.722, 0.837)		0.786 (0.730, 0.846)	
5	579/27946	359687.58	0.673 (0.606, 0.746)		0.740 (0.667, 0.822)		0.749 (0.675, 0.832)	
Non-toxic goiter								
0-1	210/31568	409976.70	Ref	*<0.001*	Ref	*0.006*	Ref	*0.010*
2	641/114868	1493721.21	0.848 (0.723, 0.994)	*-*	0.863 (0.736, 1.012)	*-*	0.867 (0.739, 1.016)	*-*
3	950/188986	2456085.81	0.759 (0.651, 0.885)		0.783 (0.672, 0.914)		0.789 (0.676, 0.921)	
4	632/131678	1709315.89	0.743 (0.632, 0.872)		0.772 (0.656, 0.907)		0.779 (0.662, 0.916)	
5	121/28998	376312.67	0.700 (0.558, 0.879)		0.733 (0.583, 0.922)		0.742 (0.590, 0.933)	
Thyrotoxicosis[Hyperthyroidism]								
0-1	255/31403	407496.91		*<0.001*	Ref	*<0.001*	Ref	*0.001*
2	756/114230	1484870.41	0.813 (0.704, 0.940)	*-*	0.850 (0.735, 0.982)	*-*	0.859 (0.744, 0.993)	*-*
3	1077/188109	2443989.90	0.712(0.619, 0.819)		0.766(0.666, 0.882)		0.780 (0.677, 0.897)	
4	714/131092	1701325.79	0.692(0.597, 0.801)		0.763(0.658, 0.885)		0.779 (0.672, 0.904)	
5	123/28894	374824.67	0.594(0.477, 0.738)		0.663(0.533, 0.826)		0.680 (0.546, 0.847)	
Thyroiditis								
0-1	32/31728	413081.09		*0.313*	Ref	*0.289*	Ref	*0.293*
2	107/115355	1503466.1	0.888(0.595, 1.326)	*-*	0.887(0.594, 1.326)	*-*	0.893 (0.597, 1.334)	*-*
3	222/189850	2472062.99	1.057(0.724, 1.544)		1.057(0.722, 1.547)		1.066 (0.728, 1.561)	
4	129/132180	1719043.61	0.857(0.577, 1.275)		0.855(0.573, 1.274)		0.863 (0.578, 1.289)	
5	28/29098	378121.93	0.849(0.507, 1.423)		0.832(0.495, 1.398)		0.841 (0.500, 1.415)	
Thyroid cancer								
0-1	33/31736	403397.53	Ref	*0.763*	Ref	*0.773*	Ref	*0.823*
2	124/115476	1482072.4	1.044 (0.707, 1.542)	*-*	1.053 (0.712, 1.556)	*-*	1.065 (0.721, 1.575)	*-*
3	187/189989	2448375.41	0.959 (0.657, 1.400)		0.968 (0.662, 1.416)		0.987 (0.675, 1.444)	
4	118/132312	1710896.01	0.877 (0.590, 1.302)		0.885 (0.594, 1.318)		0.907 (0.608, 1.352)	
5	27/29129	377098.65	0.942 (0.561, 1.580)		0.945 (0.562, 1.590)		0.973 (0.578, 1.639)	
Iodine-deficiency-related thyroid disorders and allied conditions								
0-1	1/31769	413830.9	Ref	*0.731*	Ref	*0.739*	Ref	*0.740*
2	8/115552	1506770.11	2.098 (0.262, 16827)	*-*	2.165 (0.270, 19.380)	*-*	2.182 (0.272, 17.526)	*-*
3	7/190128	2477085.26	1.092 (0.133, 8.953)		1.135 (0.138, 9.325)		1.154 (0.140, 9.492)	
4	7/132414	1722915.06	1.572 (0.191, 12.960)		1.624 (0.196, 13.431)		1.661 (0.200, 13.773)	
5	2/29144	378894.89	2.201 (0.939, 1.110)		2.206 (0.196, 24.780)		2.268 (0.201, 25.546)	
Subclinical iodine-deficiency hypothyroidism								
0-1	11/31772	413819.65	Ref	*0.030*	Ref	*0.0483*	Ref	*0.048*
2	34/115560	1506792.76	0.833 (0.421, 1.647)	*-*	0.888 (0.449, 1.756)	*-*	0.887 (0.448, 1.756)	*-*
3	75/190141	2476980.16	1.094 (0.578, 2.072)		1.188 (0.625, 2.258)		1.188 (0.625, 2.260)	
4	25/132431	1723102.35	0.508 (0.246, 1.050)		0.575 (0.277, 1.191)		0.573 (0.276, 1.191)	
5	7/29144	378904.45	0.776 (0.299, 2.015)		0.894 (0.343, 2.330)		0.893 (0.342, 2.332)	
Other disorders of thyroid								
0-1	84/31662	412439.95		*0.001*		*0.001*		*0.001*
2	264/115178	1501955.06	0.842 (0.658, 1.077)	*-*	0.857 (0.669, 1.097)	*-*	0.863 (0.674, 1.105)	*-*
3	353/189493	2468885.3	0.699 (0.555, 0.889)		0.700 (0.555, 0.891)		0.708 (0.555, 0.901)	
4	209/131964	1717116.15	0.629 (0.487, 0.813)		0.621 (0.479, 0.804)		0.628 (0.485, 0.814)	
5	52/29052	377722.76	0.807 (0.569, 1.145)		0.794 (0.559, 1.128)		0.803 (0.565, 1.142)	

HR, hazard ratio; CI, confidence interval; Model 1 was adjusted for age, sex, race, hip and BMI;Model 2 was model 1 with additional adjustments for income, education, Townsend. index, smoking and drinking;Model 3 was model 2 with additional PAL.level and diabetes baseline.The reference group consisted of participants with a healthy sleep score of 0-1.

### 3.3 Association between individual aspect of sleeping behavior and risk of developing thyroid disease

The association between individual sleep behaviors and the risk of developing thyroid disease is presented in **Figure 1**. Compared with participants whose sleep duration was shorter than 7 hours or longer than 8 hours per day, participants with a sleep duration of 7 to 8 hours per day had a lower risk of incident overall thyroid disease (HR: 0.903, 95% CI: 0.873 to 0.934), hypothyroidism (HR: 0.899, 95% CI: 0.867 to 0.933), and thyrotoxicosis (HR: 0.871, 95% CI: 0.807 to 0.940). Meanwhile, in terms of chronotype, compared with evening types, morning types had a lower risk of overall thyroid disease (HR: 0.923, 95% CI: 0.891 to 0.956) and hypothyroidism (HR: 0.916, 95% CI: 0.881 to 0.952). Furthermore, compared with participants with frequent insomnia, those without frequent insomnia had a lower risk of incident overall thyroid disease (HR: 0.931, 95% CI: 0.893 to 0.969), hypothyroidism (HR: 0.936, 95% CI: 0.895 to 0.979), nontoxic goiter (HR: 0.898, 95% CI: 0.811 to 0.994), and thyrotoxicosis (HR: 0.907, 95% CI: 0.825 to 0.997). Moreover, compared with participants with excessive daytime sleepiness, those without excessive daytime sleepiness had a lower risk of incident overall thyroid disease (HR: 0.795, 95% CI: 0.729 to 0.866), hypothyroidism (HR: 0.821, 95% CI: 0.747 to 0.902), nontoxic goiter (HR: 0.789, 95% CI: 0.645 to 0.966), and thyrotoxicosis (HR: 0.612, 95% CI: 0.518 to 0.722). Lastly, no significant association between snoring behavior and the risk of any type of thyroid disease was observed.

**Figure 1:**
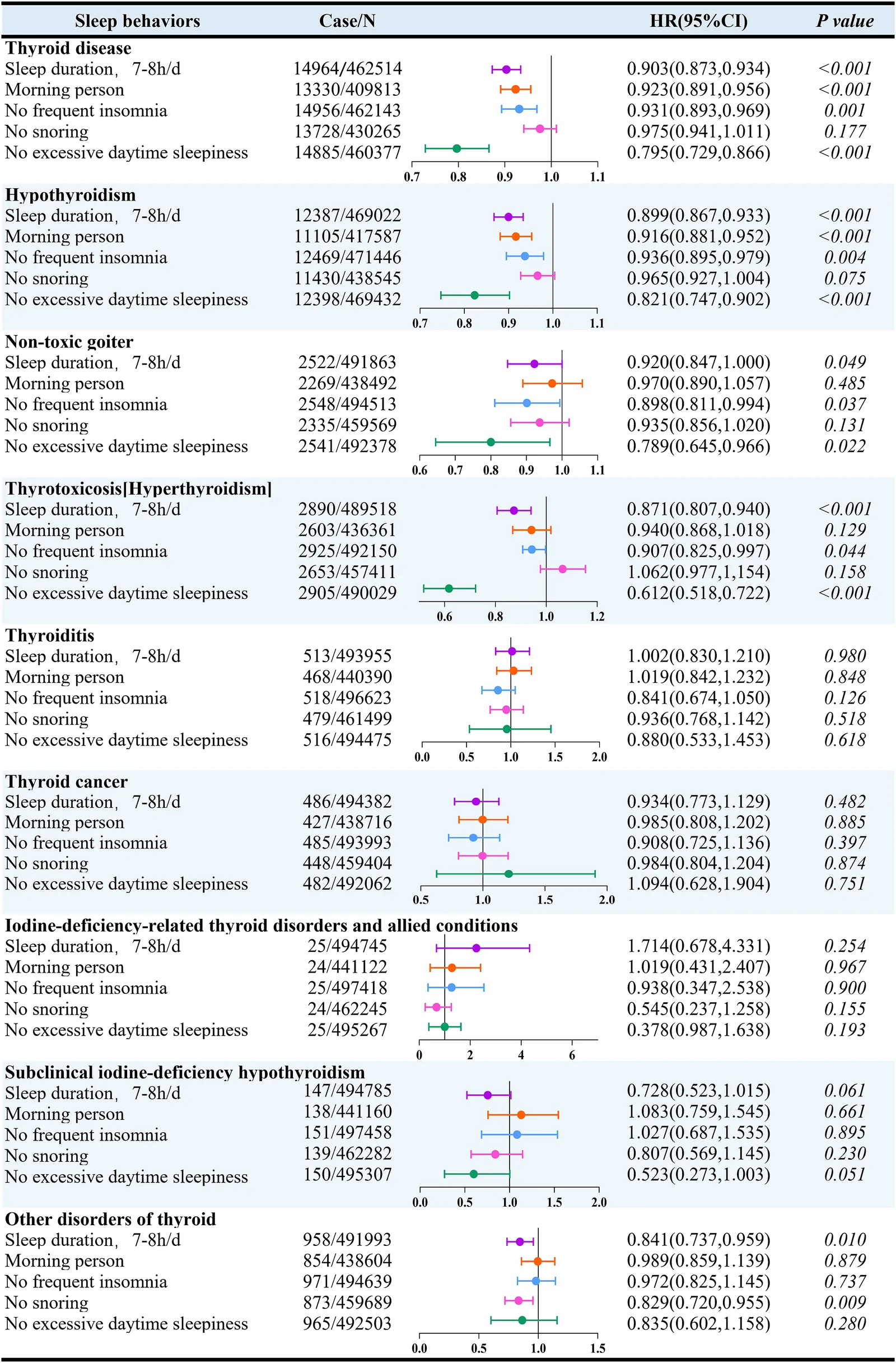
Association of individual sleep behaviors with the risk of developing overall and specific thyroid diseases. Hazard ratios (HRs) and 95% confidence intervals (CIs) were calculated using Cox proportional hazards regression models. The models evaluated five individual sleep behaviors: sleep duration (7–8 hours/day vs. others), chronotype (morning vs. evening), insomnia (no frequent insomnia vs. frequent), daytime sleepiness (no frequent excessive sleepiness vs. excessive), and snoring (no vs. yes). Results were adjusted for covariates including demographic factors, lifestyle behaviors, PAL Level and preexisting diabetes (Model 3).

### 3.4 Association between healthy sleep scores and the risk of incident thyroid diseases in individuals with different characteristics

**Figure 2** presents the associations between healthy sleep scores and the risk of incident overall thyroid disease across different population characteristics. Interaction analyses indicated that the association between healthy sleep scores and the risk of overall thyroid disease differed by sex (*P*_interaction_<0.001). Compared with participants with low healthy sleep scores, those with a healthy sleep score of 5 had a lower risk of overall thyroid disease in both women and men, with HRs (95% CIs) of 0.730 (0.652–0.817) and 0.793 (0.666–0.944), respectively; the inverse association was more pronounced among women. In addition, BMI (*P*_interaction_ < 0.05), diabetes status ((*P*_interaction_ *<*0.05), and former smoking status ((*P*_interaction_=0.001) significantly modified the association between healthy sleep scores and the risk of overall thyroid disease. A healthy sleep score of 5 was associated with a lower risk of overall thyroid disease among overweight (HR = 0.714, 95% CI: 0.613–0.832) and obese participants (HR = 0.703, 95% CI: 0.580–0.852), as well as among participants without diabetes (HR = 0.760, 95% CI: 0.688–0.840). For smoking status, a healthy sleep score of 5 was associated with a lower risk of overall thyroid disease among never smokers (HR = 0.784, 95% CI: 0.689–0.893), former smokers (HR = 0.733, 95% CI: 0.622–0.865), and current smokers (HR = 0.717, 95% CI: 0.531–0.967); however, only the interaction for former smoking status was statistically significant. No significant effect modification was observed for age (*P*_interaction_=0.908), former alcohol consumption (*P*_interaction_=0.174), or current alcohol consumption (*P*_interaction_ =0.117) in the association between healthy sleep scores and the risk of overall thyroid disease. Therefore, although the effect estimates varied slightly across age and alcohol consumption subgroups, there was no statistical evidence that this association differed by alcohol consumption status.

**Figure 2:**
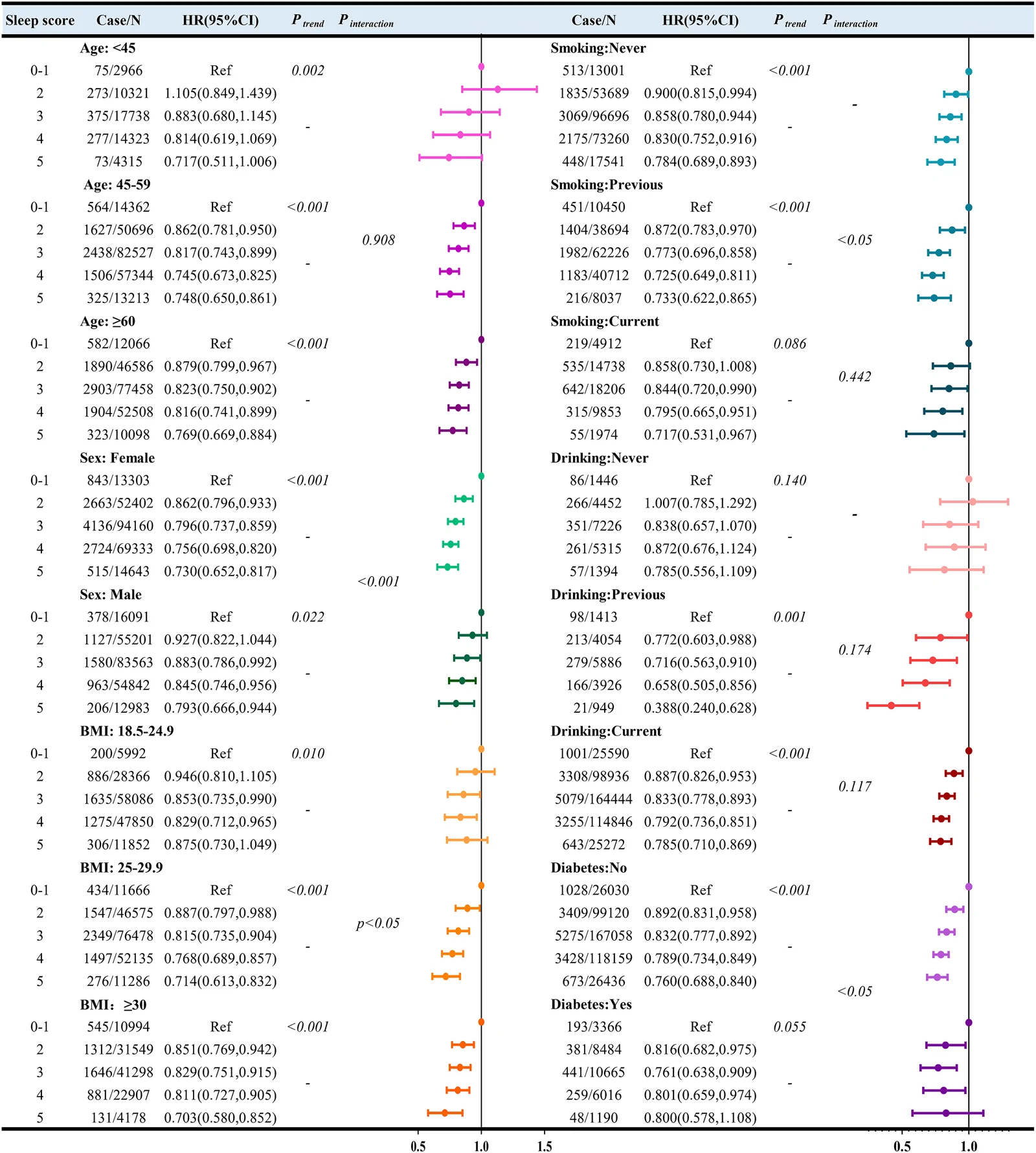
Stratified analyses of the association between healthy sleep score and the risk of incident overall thyroid diseases. Hazard ratios (HRs) and 95% confidence intervals (CIs) compare individuals with a healthy sleep score of 5 against those with a score of 0–1 across various subgroups.

For hypothyroidism, nontoxic goiter, and thyrotoxicosis, for which statistically significant associations between healthy sleep scores and disease risk were observed in the stratified analyses, interaction tests were further conducted (**Supplementary Figures 1–3**). The association between healthy sleep scores and the risk of hypothyroidism appeared to be modified by age, former alcohol consumption, and current alcohol consumption (*P*_interaction_ *<*0.05). In contrast, no statistically significant interactions were observed for nontoxic goiter or thyrotoxicosis *P*_interaction_ *>*0.05).

Exploratory stratified analyses were conducted for thyroiditis, thyroid cancer, iodine-deficiency-related thyroid disorders and allied conditions, iodine-deficiency hypothyroidism, and other thyroid disorders; no statistically significant dose–response associations between healthy sleep scores and the risk of the corresponding diseases were observed across subgroups (**Supplementary Figures 4–8**).

### 3.5 Sensitivity analysis

After excluding participants with less than 1 year of follow-up or incident thyroid disease within the first year of follow-up (**Supplementary Table 2**), healthy sleep scores were inversely associated with the risk of thyroid disease, hypothyroidism, nontoxic goiter, and thyrotoxicosis in a dose-dependent manner. Stratified analyses showed no significant association between snoring and the risk of incident thyroid disease. Therefore, we re-evaluated the sleep score after removing snoring as a sleep behavior. The re-evaluated sleep score was inversely associated with the risk of the above diseases in a dose-dependent manner (**Supplementary Table 3**).

## 4 Discussion

In this study, higher healthy sleep scores were associated with a lower risk of incident thyroid disease, including hypothyroidism, nontoxic goiter, and thyrotoxicosis. In addition, sleep duration, chronotype, insomnia frequency, and daytime excessive sleepiness were associated with the risk of certain thyroid diseases, whereas no significant association was observed between snoring and the risk of any thyroid disease. Furthermore, the association between healthy sleep scores and the risk of overall thyroid disease may vary by sex, BMI, diabetes status, and smoking status.

Currently, studies on the health effects of sleep behaviors on the development and progression of thyroid diseases have mainly focused on sleep duration or quality in cross-sectional settings. Although shorter sleep duration or poorer sleep quality has been associated with disrupted thyroid hormone levels or subclinical hypothyroidism (13–15), the association between sleep behaviors and the future risk of incident thyroid disease remains unclear. Compared with previous studies, our study provides further prospective evidence for the association between sleep behaviors and the risk of incident thyroid disease. We found that higher healthy sleep scores were associated with a lower risk of incident thyroid disease, including hypothyroidism, nontoxic goiter, and thyrotoxicosis. It is well known that TSH is influenced by circadian rhythms (6). Through mechanisms similar to those underlying dysregulated TSH rhythms, circadian disruption has also been associated with thyroid cancer (16). Alterations in sleep architecture may activate the hypothalamic–pituitary axis and affect TSH levels (17). Animal studies have shown that sleep deprivation increases prostaglandin D2 (PGD2) levels in the brain (18), which may affect thyroid hormone activity (19). On the other hand, autoimmune mechanisms may contribute to the development and progression of certain thyroid diseases, such as some forms of hypothyroidism, thyroiditis, and thyrotoxicosis. Studies have found that sleep deprivation alters the composition of immune cells in mice and exacerbates autoimmune responses by inducing pathological immune cell activation, upregulating inflammatory pathways, and promoting intercellular communication (20). Therefore, immune and inflammatory pathways may provide potential explanations for the observed associations between sleep and certain thyroid diseases.

Furthermore, healthy sleep scores generally incorporate several components, including sleep duration, chronotype, insomnia frequency, daytime excessive sleepiness, and snoring. We therefore examined whether these behaviors were associated with thyroid diseases and whether their associations differed across specific types of thyroid disease. Among the five sleep components, the absence of frequent daytime excessive sleepiness showed more apparent associations with a lower risk of several thyroid diseases. However, unlike sleep duration and chronotype, daytime excessive sleepiness is primarily a subjective symptom rather than a sleep behavior alone. It may reflect insufficient sleep or poor sleep quality and may also be associated with underlying health conditions, including obesity, depression, neurological disorders, medication use, and abnormal glucose metabolism, which may themselves be associated with the risk of thyroid disease. Therefore, despite adjustment for multiple potential confounders, the association between daytime excessive sleepiness and the risk of thyroid disease may still be affected by residual confounding. Animal studies have suggested that sleepiness is associated with hypocretin (Hcrt) deficiency (21). Hcrt deficiency may affect the neuroendocrine system, leading to dysregulation of energy balance, feeding behavior, and glucose metabolism (22), and may also regulate the hypothalamic–pituitary–thyroid axis in rodents (23). This may be one of the underlying factors linking daytime sleepiness to the risk of incident thyroid disease. In addition, we found that snoring was not associated with the major types of thyroid disease and was associated only with an increased risk of other thyroid disorders, suggesting that no significant association was observed between snoring and the risk of incident thyroid disease in most categories. Future studies should further investigate specific diseases covered by ICD-10 code E07 to clarify the associations of snoring and other sleep-disordered breathing characteristics with thyroid disease risk. In addition to snoring, more severe sleep-disordered breathing may have a substantial effect on thyroid function. A recent study showed that, among patients with obstructive sleep apnea, apnea-specific hypoxic burden and the sleep-disordered breathing index were independently associated with alterations in thyroid function, suggesting that the severity of nocturnal hypoxic burden may better reflect sleep-disordered breathing characteristics associated with thyroid function alterations than snoring itself (24).

To further assess whether the association between healthy sleep scores and the risk of overall thyroid disease differed by population characteristics, we conducted subgroup analyses and tested for interactions. The association between healthy sleep scores and the risk of overall thyroid disease may vary by sex, BMI, diabetes status, and former smoking status. Thyroid diseases, particularly autoimmune thyroid diseases, are more common in women, and sex hormone levels, X chromosome-related immune regulation, and a greater predisposition to autoimmune responses may all contribute to their development and progression. Insufficient sleep, insomnia, and circadian rhythm disruption may affect TSH secretion through the hypothalamic–pituitary–thyroid axis and subsequently influence inflammatory responses and immune homeostasis. Therefore, the sex heterogeneity observed in this study may reflect the combined effects of sleep behaviors and sex-specific endocrine and immune regulation. The metabolic status represented by BMI may also influence this association. Overweight and obesity are commonly accompanied by insufficient sleep duration, poor sleep quality, circadian rhythm disruption, and sleep-disordered breathing, and may affect thyroid function through lipotoxicity, adipokine imbalance, and increased secretion of inflammatory cytokines (25, 26). Obesity-related chronic low-grade inflammation, insulin resistance, and abnormal leptin signaling may further disrupt the hypothalamic–pituitary–thyroid axis, making the relationship between sleep behaviors and thyroid disease more complex. Diabetes and thyroid disease may share some genetic and immunological bases. Previous studies have suggested that genetic variants in HLA-DR, PTPN22, CTLA-4, and FOXP3 may jointly contribute to the development of diabetes and thyroid disease (27, 28). In addition, diabetes-related insulin resistance, chronic inflammation, oxidative stress, and elevated leptin levels may alter TSH synthesis and secretion by affecting the hypothalamic–pituitary–thyroid axis (29). Diabetes is also commonly accompanied by sleep disturbances, suggesting potential interrelationships among sleep, abnormal glucose metabolism, and thyroid function.

## 5 Strength and limitation

This study has several limitations. First, sleep behaviors were assessed only at baseline through self-report, which may be subject to recall bias and subjective reporting errors, resulting in misclassification. In addition, given the long follow-up period, sleep behaviors may have changed over time, and baseline measurements could not capture such dynamic changes during follow-up, potentially leading to further exposure misclassification. Second, although multiple potential confounders were adjusted for, residual confounding due to obstructive sleep apnea, mental health status, medication use, and other unmeasured factors cannot be excluded. Moreover, undiagnosed subclinical thyroid dysfunction may have already affected sleep status; therefore, reverse causality cannot be completely ruled out. Third, thyroid disease outcomes were ascertained using ICD codes from medical records, which may have resulted in outcome misclassification. Participants with more sleep problems or greater health awareness may have undergone more medical evaluations, potentially leading to detection bias. Fourth, baseline information on the use of thyroid-related medications was not available. Although participants with pre-existing thyroid disease were excluded based on baseline diagnostic records, some previously treated cases without identifiable diagnostic codes may still have been misclassified as incident cases during follow-up, potentially resulting in some degree of outcome misclassification.

## 6 Conclusion

Higher healthy sleep scores were associated with lower risks of incident overall thyroid disease, hypothyroidism, nontoxic goiter, and thyrotoxicosis. Among the five sleep components, the absence of frequent daytime excessive sleepiness showed the most apparent associations with lower risks of these diseases. Interaction analyses suggested that the association between healthy sleep scores and the risk of overall thyroid disease was more pronounced among women, participants with obesity, and those without diabetes. Further intervention and causal inference studies are needed to determine whether improving sleep can reduce the risk of thyroid disease.

## Data Availability

The datasets presented in this article are not readily available because The data used in this study are available from the UK Biobank (https://www.ukbiobank.ac.uk/) under application number 103547, upon application and subject to the UK Biobank’s data access procedures and approval. Due to the UK Biobank’s data-sharing and licensing policies, the data cannot be publicly deposited or directly shared by the authors. Requests to access the datasets should be directed to https://www.ukbiobank.ac.uk/.
