# Supplementary material for: Healthy sleep pattern is associated with reduced risk of incident thyroid disease: a prospective cohort study from the UK Biobank
Source: Front Endocrinol (Lausanne). 2026 Oct 2;17:1859828. doi: 10.3389/fendo.2026.1859828 (PMC13672026; doi:10.3389/fendo.2026.1859828)
Supplement: Supplementary file 1 [file DataSheet1.docx]

Supplementary Material

Supplementary Figure 1. Stratified analyses of the association between healthy sleep score and the risk of incident hypothyroidism. Hazard ratios (HRs) and 95% confidence intervals (CIs) for incident hypothyroidism are presented for participants with different healthy sleep scores (reference: score 0-1) across subgroups categorized by age, gender, BMI, smoking status, drinking status, and preexisting diabetes.

Supplementary Figure 2. Stratified analyses of the association between healthy sleep score and the risk of incident non-toxic goiter. Hazard ratios (HRs) and 95% confidence intervals (CIs) for incident non-toxic goiter are presented for participants with different healthy sleep scores (reference: score 0-1) across subgroups categorized by age, gender, BMI, smoking status, drinking status, and preexisting diabetes.

Supplementary Figure 3. Stratified analyses of the association between healthy sleep score and the risk of incident thyrotoxicosis. Hazard ratios (HRs) and 95% confidence intervals (CIs) for incident thyrotoxicosis are presented for participants with different healthy sleep scores (reference: score 0-1) across subgroups categorized by age, gender, BMI, smoking status, drinking status, and preexisting diabetes.

Supplementary Figure 4. Stratified analyses of the association between healthy sleep score and the risk of incident thyroiditis. Hazard ratios (HRs) and 95% confidence intervals (CIs) for incident thyroiditis are presented for participants with different healthy sleep scores (reference: score 0-1) across subgroups categorized by age, gender, BMI, smoking status, drinking status, and preexisting diabetes.

Supplementary Figure 5. Stratified analyses of the association between healthy sleep score and the risk of incident thyroid cancer. Hazard ratios (HRs) and 95% confidence intervals (CIs) for incident thyroid cancer are presented for participants with different healthy sleep scores (reference: score 0-1) across subgroups categorized by age, gender, BMI, smoking status, drinking status, and preexisting diabetes.

Supplementary Figure 6. Stratified analyses of the association between healthy sleep score and the risk of incident iodine deficiency related thyroid diseases and related diseases. Hazard ratios (HRs) and 95% confidence intervals (CIs) for incident iodine deficiency related thyroid diseases and related diseases are presented for participants with different healthy sleep scores (reference: score 0-1) across subgroups categorized by age, gender, BMI, smoking status, drinking status, and preexisting diabetes.

Supplementary Figure 7. Stratified analyses of the association between healthy sleep score and the risk of incident subclinical iodine deficiency hypothyroidism. Hazard ratios (HRs) and 95% confidence intervals (CIs) for incident subclinical iodine deficiency hypothyroidism are presented for participants with different healthy sleep scores (reference: score 0-1) across subgroups categorized by age, gender, BMI, smoking status, drinking status, and preexisting diabetes.

Supplementary Figure 8. Stratified analyses of the association between healthy sleep score and the risk of incident other thyroid diseases. Hazard ratios (HRs) and 95% confidence intervals (CIs) for incident other thyroid diseases are presented for participants with different healthy sleep scores (reference: score 0-1) across subgroups categorized by age, gender, BMI, smoking status, drinking status, and preexisting diabetes.

Supplementary Table 1. The number of risk sets for thyroid disease outcomes and the number of new events during follow-up in different healthy sleep scoring groups.

| **Disease** | **Healthy Sleep Score** | | | | | ***P value*** |
| --- | --- | --- | --- | --- | --- | --- |
|  | **0-1** | **2** | **3** | **4** | **5** |  |
| **Thyroid disease[n(%)]** | | | | | | |
| No | 28175(88.7) | 103814(89.8) | 172007(90.5) | 120488(91) | 26905(92.3) | ＜0.001 |
| Yes* | 1221(3.8) | 3790(3.3) | 5716(3) | 3687(2.8) | 721(2.5) | - |
| **Hypothyroidism[n(%)]** | | | | | | |
| No | 28817（90.7） | 105949（91.7） | 175546（92.3） | 122872（92.8） | 27367（93.9） | ＜0.001 |
| Yes | 1028（3.2） | 3159（2.7） | 4750（2.5） | 3021（2.3） | 579（2） | - |
| **Non-toxic goiter[n(%)]** | | | | | | |
| No | 31358（98.7） | 114227（98.8） | 188036（98.9） | 131046（99） | 28877（99.1） | ＜0.001 |
| Yes* | 210（0.7） | 641（0.6） | 950（0.5） | 632（0.5） | 121（0.4） | - |
| **Thyrotoxicosis[n(%)]** | | | | | | |
| No | 31148（98） | 113474（98.2） | 187032（98.4） | 130378（98.4） | 28771（98.7） | ＜0.001 |
| Yes* | 255（0.8） | 756（0.7） | 1077（0.6） | 714（0.5） | 123（0.4） | - |
| **Thyroiditis[n(%)]** | | | | | | |
| No | 31696（99.8） | 115248（99.7） | 189628（99.7） | 132051（99.7） | 29070（99.7） | 0.179 |
| Yes* | 32（0.1） | 107（0.1） | 222（0.1） | 129（0.1） | 28（0.1） | - |
| **Thyroid cancer[n(%)]** | | | | | | |
| No | 31703(99.8) | 115352(99.8) | 189802(99.8) | 132194(99.8) | 29102(99.9) | 0.181 |
| Yes* | 33(0.1) | 124(0.1) | 187(0.1) | 118(0.1) | 27(0.1) |  |
| **Iodine-deficiency-related thyroid disorders and allied conditions[n(%)]** | | | | | | |
| No | 31768（100） | 115544（100） | 190121（100） | 132407（100） | 29142（100） | 0.734 |
| Yes* | 1（0） | 8（0） | 7（0） | 7（0） | 2（0） | - |
| **Subclinical iodine-deficiency hypothyroidism[n(%)]** | | | | | | |
| No | 31761（100） | 115526（100） | 190066（100） | 132406（100） | 29137（100） | 0.024 |
| Yes* | 11（0） | 34（0） | 75（0） | 25（0） | 7（0） | - |
| **Other disorders of thyroid[n(%)]** | | | | | | |
| No | 31578(99.4) | 114914(99.4) | 189140(99.5) | 131755(99.5) | 29000(99.5) | 0.001 |
| Yes* | 84(0.3) | 264(0.2) | 353(0.2) | 209(0.2) | 52(0.2) | - |

Supplementary Table 2. Sensitivity analysis for the association between healthy sleep score and the risk of thyroid diseases after excluding events occurring within the first year of follow-up.

| **Sleep score** | **Cases/N** | **Model1** | | **Model2** | | **Model3** | |
| --- | --- | --- | --- | --- | --- | --- | --- |
|  |  | **HR(95%CI)** | ***P value*** | **HR(95%CI)** | ***P value*** | **HR(95%CI)** | ***P value*** |
| **Thyroid disease** |  |  |  |  |  |  |  |
| 0-1 | 1151/29218 | Ref | *<0.001* | Ref | *<0.001* | Ref | *<0.001* |
| 2 | 3574/107125 | 0.849(0.794,0.909) | *-* | 0.878(0.820,0.940) | *-* | 0.885(0.827,0.948) | *-* |
| 3 | 5435/177095 | 0.776(0.727,0.828) |  | 0.823(0.770,0.879) |  | 0.833(0.780,0.890) |  |
| 4 | 3498/123780 | 0.725(0.677,0.776) |  | 0.783(0.731,0.839) |  | 0.795(0.742,0.852) |  |
| 5 | 686/27543 | 0.692(0.629,0.762) |  | 0.756(0.686,0.833) |  | 0.769(0.698,0.847) |  |
| **Hypothyroidism** |  |  |  |  |  |  |  |
| 0-1 | 959/29776 | Ref | *<0.001* | Ref | *<0.001* | Ref | *<0.001* |
| 2 | 2984/108932 | 0.860(0.798,0.926) | ***-*** | 0.891(0.827,0.960) | ***-*** | 0.897(0.832,0.966) | ***-*** |
| 3 | 4505/180051 | 0.783(0.729,0.842) |  | 0.835(0.777,0.898) |  | 0.843(0.784,0.907) |  |
| 4 | 2860/125731 | 0.724(0.671,0.781) |  | 0.788(0.730,0.851) |  | 0.797(0.738,0.860) |  |
| 5 | 550/27917 | 0.683(0.614,0.761) |  | 0.753(0.676,0.839) |  | 0.762(0.685,0.849) |  |
| **Non-toxic goiter** |  |  |  |  |  |  |  |
| 0-1 | 195/31553 | Ref | *0.005* | Ref | *0.032* | Ref | *0.046* |
| 2 | 591/114817 | 0.847(0.718,0.999) | *-* | 0.863(0.731,1.018) | *-* | 0.866(0.734,1.022) | *-* |
| 3 | 888/188924 | 0.772(0.658,0.905) |  | 0.798(0.680,0.937) |  | 0.803(0.684,0.943) |  |
| 4 | 593/131638 | 0.761(0.644,0.899) |  | 0.794(0.671,0.939) |  | 0.800(0.676,0.947) |  |
| 5 | 112/28989 | 0.709(0.560,0.898) |  | 0.744(0.587,0.943) |  | 0.753(0.593,0.955) |  |
| **Thyrotoxicosis[Hyperthyroidism]** |  |  |  |  |  |  |  |
| 0-1 | 254/31391 | Ref | *<0.001* | Ref | *<0.001* | Ref | *0.001* |
| 2 | 752/114179 | 0.812(0.703,0.939) | ***-*** | 0.848(0.734,0.980) | ***-*** | 0.858(0.742,0.992) | ***-*** |
| 3 | 1076/188050 | 0.714(0.621,0.821) |  | 0.768(0.667,0.884) |  | 0.781(0.678,0.899) |  |
| 4 | 711/131056 | 0.691(0.596,0.800) |  | 0.762(0.657,0.884) |  | 0.778(0.670,0.902) |  |
| 5 | 121/28885 | 0.586(0.470,0.729) |  | 0.654(0.525,0.816) |  | 0.670(0.537,0.836) |  |
| **Thyroiditis** |  |  |  |  |  |  |  |
| 0-1 | 30/31726 | Ref | *0.247* | Ref | *0.222* | Ref | *0.223* |
| 2 | 99/115346 | 0.882(0.583,1.336) | *-* | 0.878(0.579,1.330) | *-* | 0.882(0.582,1.337) | *-* |
| 3 | 209/189837 | 1.070(0.723,1.583) |  | 1.066(0.719,1.580) |  | 1.072(0.723,1.590) |  |
| 4 | 121/132171 | 0.867(0.575,1.307) |  | 0.860(0.569,1.300) |  | 0.866(0.572,1.310) |  |
| 5 | 24/29094 | 0.784(0.454,1.354) |  | 0.764(0.441,1.323) |  | 0.768(0.443,1.332) |  |
| **Thyroid cancer** |  |  |  |  |  |  |  |
| 0-1 | 31/31619 | Ref | *0.72* | Ref | *0.748* | Ref | *0.809* |
| 2 | 115/115186 | 1.027(0.686,1.538) | *-* | 1.039(0.694,1.556) | *-* | 1.053(0.703,1.578) | *-* |
| 3 | 176/189604 | 0.957(0.648,1.414) |  | 0.971(0.656,1.437) |  | 0.994(0.671,1.472) |  |
| 4 | 108/132078 | 0.850(0.564,1.280) |  | 0.864(0.572,1.305) |  | 0.889(0.588,1.345) |  |
| 5 | 25/29077 | 0.925(0.541,1.580) |  | 0.934(0.545,1.602) |  | 0.968(0.564,1.662) |  |
| **Iodine-deficiency-related thyroid disorders and allied conditions** |  |  |  |  |  |  |  |
| 0-1 | 1/31769 | Ref | *0.604* | Ref | *0.611* | Ref | *0.592* |
| 2 | 8/115551 | 2.112(0.263,16.942) | ***-*** | 2.278(0.282,18.368) | ***-*** | 2.264(0.278,18.408) | ***-*** |
| 3 | 6/190127 | 0.948(0.113,7.945) |  | 1.046(0.124,8.832) |  | 0.997(0.117,8.497) |  |
| 4 | 6/132412 | 1.371(0.163,11.557) |  | 1.539(0.181,13.113) |  | 1.426(0.166,12.275) |  |
| 5 | 2/29144 | 2.240(0.200,25.103) |  | 2.490(0.220,28.188) |  | 2.220(0.194,25.392) |  |
| **Subclinical iodine-deficiency hypothyroidism** |  |  |  |  |  |  |  |
| 0-1 | 11/31772 | Ref | *0.043* | Ref | *0.069* | Ref | *0.067* |
| 2 | 34/115559 | 0.834(0.422,1.648) | *-* | 0.883(0.446,1.748) | *-* | 0.883(0.446,1.748) | *-* |
| 3 | 73/190139 | 1.064(0.561,2.018) |  | 1.147(0.603,2.182) |  | 1.146(0.602,2.183) |  |
| 4 | 25/132430 | 0.508(0.246,1.05) |  | 0.569(0.275,1.181) |  | 0.568(0.273,1.179) |  |
| 5 | 7/29144 | 0.775(0.298,2.014) |  | 0.884(0.339,2.306) |  | 0.882(0.337,2.304) |  |
| **Other disorders of thyroid** |  |  |  |  |  |  |  |
| 0-1 | 84/31662 | Ref | *0.001* | Ref | *0.001* | Ref | *0.001* |
| 2 | 264/115177 | 0.842(0.658,1.077) | ***-*** | 0.857(0.669,1.097) | ***-*** | 0.863(0.674,1.105) | ***-*** |
| 3 | 353/189493 | 0.699(0.550,0.889) |  | 0.700(0.550,0.891) |  | 0.708(0.555,0.901) |  |
| 4 | 209/131963 | 0.629(0.487,0.813) |  | 0.621(0.479,0.804) |  | 0.628(0.485,0.814) |  |
| 5 | 52/29052 | 0.807(0.569,1.145) |  | 0.794(0.559,1.128) |  | 0.803(0.565,1.142) |  |

Note: HR, hazard ratio; CI, confidence interval. Model 1 was adjusted for age, sex, race, hip and BMI;Model 2 was model 1 with additional adjustments for income, education, Townsend. index, smoking and drinking;Model 3 was model 2 with additional PAL .level and diabetes baseline.The reference group consisted of participants with a healthy sleep score of 0-1.

Supplementary Table 3. Sensitivity analysis for the association between healthy sleep score and the risk of thyroid diseases after removing snoring as a sleeping behavior.

| **Sleep score** | **Cases/N** | **Model1** | | **Model2** | | **Model3** | |
| --- | --- | --- | --- | --- | --- | --- | --- |
|  |  | **HR(95%CI)** | ***P value*** | **HR(95%CI)** | ***P value*** | **HR(95%CI)** | ***P value*** |
| **Thyroid disease** |  |  |  |  |  |  |  |
| 0-1 | 2511/61774 | Ref | *<0.001* | Ref | *<0.001* | Ref | *<0.001* |
| 2 | 6042/179890 | 0.831(0.792,0.871) | *-* | 0.862(0.823,0.904) | *-* | 0.868(0.828,0.911) | *-* |
| 3 | 5398/177606 | 0.768(0.732,0.806) |  | 0.818(0.779,0.859) |  | 0.826(0.787,0.868) |  |
| 4 | 1184/47254 | 0.712(0.664,0.764) |  | 0.768(0.716,0.824) |  | 0.779(0.726,0.835) |  |
| **Hypothyroidism** |  |  |  |  |  |  |  |
| 0-1 | 2113/62749 | Ref | *<0.001* | Ref | *<0.001* | Ref | *<0.001* |
| 2 | 5055/182576 | 0.837(0.795,0.881) | ***-*** | 0.873(0.829,0.920) | ***-*** | 0.878(0.834,0.925) | ***-*** |
| 3 | 4383/179982 | 0.756(0.717,0.797) |  | 0.811(0.769,0.856) |  | 0.818(0.775,0.863) |  |
| 4 | 986/47781 | 0.727(0.674,0.785) |  | 0.792(0.733,0.856) |  | 0.800(0.740,0.864) |  |
| **Non-toxic goiter** |  |  |  |  |  |  |  |
| 0-1 | 426/66450 | Ref | *0.001* | Ref | *0.005* | Ref | *0.008* |
| 2 | 1010/191892 | 0.840(0.749,0.942) | *-* | 0.857(0.763,0.961) | *-* | 0.860(0.766,0.965) | *-* |
| 3 | 931/188331 | 0.824(0.733,0.925) |  | 0.850(0.756,0.956) |  | 0.855(0.760,0.962) |  |
| 4 | 187/49425 | 0.717(0.603,0.854) |  | 0.746(0.627,0.889) |  | 0.753(0.632,0.897) |  |
| **Thyrotoxicosis[Hyperthyroidism]** |  |  |  |  |  |  |  |
| 0-1 | 532/66076 | Ref | *<0.001* | Ref | *<0.001* | Ref | *<0.001* |
| 2 | 1174/190949 | 0.784(0.706,0.87) | ***-*** | 0.820(0.739,0.910) | ***-*** | 0.829(0.747,0.920) | ***-*** |
| 3 | 1016/187442 | 0.719(0.647,0.800) |  | 0.776(0.697,0.865) |  | 0.788(0.707,0.878) |  |
| 4 | 203/49261 | 0.622(0.528,0.732) |  | 0.682(0.579,0.804) |  | 0.696(0.590,0.820) |  |
| **Thyroiditis** |  |  |  |  |  |  |  |
| 0-1 | 70/66784 | Ref | *0.146* | Ref | *0.123* | Ref | *0.125* |
| 2 | 201/192788 | 0.970(0.737,1.276) | *-* | 0.967(0.734,1.274) | *-* | 0.972(0.738,1.281) | *-* |
| 3 | 213/189045 | 1.066(0.811,1.400) |  | 1.068(0.811,1.406) |  | 1.075(0.816,1.418) |  |
| 4 | 34/49594 | 0.698(0.462,1.055) |  | 0.689(0.455,1.044) |  | 0.695(0.459,1.054) |  |
| **Thyroid cancer** |  |  |  |  |  |  |  |
| 0-1 | 71/66834 | Ref | *0.491* | Ref | *0.504* | Ref | *0.561* |
| 2 | 207/192968 | 1.021(0.777,1.340) | ***-*** | 1.030(0.784,1.353) | ***-*** | 1.041(0.792,1.369) | ***-*** |
| 3 | 169/189201 | 0.878(0.663,1.162) |  | 0.886(0.668,1.176) |  | 0.902(0.679,1.198) |  |
| 4 | 42/49639 | 0.894(0.608,1.313) |  | 0.900(0.611,1.325) |  | 0.921(0.624,1.357) |  |
| **Iodine-deficiency-related thyroid disorders and allied conditions** |  |  |  |  |  |  |  |
| 0-1 | 3/66895 | Ref | *0.939* | Ref | *0.922* | Ref | *0.948* |
| 2 | 9/193111 | 1.023(0.276,3.791) | ***-*** | 1.093(0.294,4.067) | ***-*** | 1.072(0.287,4) | ***-*** |
| 3 | 10/189341 | 1.183(0.324,4.323) |  | 1.279(0.347,4.717) |  | 1.226(0.331,4.539) |  |
| 4 | 3/49660 | 1.515(0.303,7.57) |  | 1.626(0.323,8.196) |  | 1.52(0.3,7.705) |  |
| **Subclinical iodine-deficiency hypothyroidism** |  |  |  |  |  |  |  |
| 0-1 | 25/66901 | Ref | *0.546* | Ref | *0.788* | Ref | *0.788* |
| 2 | 62/193123 | 0.829(0.519,1.323) | *-* | 0.869(0.543,1.392) | *-* | 0.869(0.542,1.392) | *-* |
| 3 | 55/189362 | 0.763(0.473,1.231) |  | 0.841(0.519,1.363) |  | 0.84(0.518,1.362) |  |
| 4 | 10/49662 | 0.608(0.291,1.27) |  | 0.689(0.329,1.445) |  | 0.689(0.328,1.447) |  |
| **Other disorders of thyroid** |  |  |  |  |  |  |  |
| 0-1 | 161/66670 | Ref | *0.016* | Ref | *0.007* | Ref | *0.009* |
| 2 | 409/192435 | 0.917(0.764,1.102) | ***-*** | 0.911(0.758,1.095) | ***-*** | 0.917(0.763,1.103) | ***-*** |
| 3 | 308/188729 | 0.759(0.626,0.920) |  | 0.738(0.608,0.896) |  | 0.745(0.613,0.905) |  |
| 4 | 84/49515 | 0.947(0.726,1.236) |  | 0.919(0.703,1.201) |  | 0.929(0.710,1.215) |  |

Note: HR, hazard ratio; CI, confidence interval. Model 1 was adjusted for age, sex, race, hip and BMI;Model 2 was model 1 with additional adjustments for income, education, Townsend. index, smoking and drinking;Model 3 was model 2 with additional PAL .level and diabetes baseline.The reference group consisted of participants with a healthy sleep score of 0-1.
